# Multi-Isotopic (*δ*^2^H, *δ*^13^C, *δ*^15^N) Tracing of Molt Origin for Red-Winged Blackbirds Associated with Agro-Ecosystems

**DOI:** 10.1371/journal.pone.0165996

**Published:** 2016-11-15

**Authors:** Scott J. Werner, Keith A. Hobson, Steven L. Van Wilgenburg, Justin W. Fischer

**Affiliations:** 1 United States Department of Agriculture, Animal and Plant Health Inspection Service, Wildlife Services, National Wildlife Research Center, 4101 LaPorte Avenue, Fort Collins, CO, United States of America, 80521; 2 Environment Canada, 11 Innovation Boulevard, Saskatoon, SK, Canada, S7N 3H5; 3 Department of Biology, University of Western Ontario, 1151 Richmond St. N., London, ON, Canada, N6A 5B7; 4 Environment Canada, Prairie and Northern Wildlife Research Center, Canadian Wildlife Service, 115 Perimeter Road, Saskatoon, SK, Canada, S7N 0X4; Universita degli Studi di Milano-Bicocca, ITALY

## Abstract

We analyzed stable-hydrogen (*δ*^2^H), carbon (*δ*^13^C) and nitrogen (*δ* ^15^N) isotope ratios in feathers to better understand the molt origin and food habits of Red-winged Blackbirds (*Agelaius phoeniceus*) near sunflower production in the Upper Midwest and rice production in the Mid-South of the United States. Outer primary feathers were used from 661 after-second-year (ASY) male blackbirds collected in Minnesota, Montana, North Dakota and South Dakota (spring collection), and Arkansas, Louisiana, Mississippi, Missouri and Texas (winter collection). The best-fit model indicated that the combination of feather *δ*^2^H, *δ*^13^C and *δ*^15^N best predicted the state of sample collections and thus supported the use of this approach for tracing molt origins in Red-winged Blackbirds. When considering only birds collected in spring, 56% of birds were classified to their collection state on the basis of *δ*^2^H and *δ*^13^C alone. We then developed feather isoscapes for *δ*^13^C based upon these data and for *δ*^2^H based upon continental patterns of *δ*^2^H in precipitation. We used 81 birds collected at the ten independent sites for model validation. The spatially-explicit assignment of these 81 birds to the *δ*^2^H isoscape resulted in relatively high rates (~77%) of accurate assignment to collection states. We also modeled the spatial extent of C3 (e.g. rice, sunflower) and C4 (corn, millet, sorghum) agricultural crops grown throughout the Upper Midwest and Mid-South United States to predict the relative use of C3- versus C4-based foodwebs among sampled blackbirds. Estimates of C3 inputs to diet ranged from 50% in Arkansas to 27% in Minnesota. As a novel contribution to blackbird conservation and management, we demonstrate how such feather isoscapes can be used to predict the molt origin and interstate movements of migratory blackbirds for subsequent investigations of breeding biology (e.g. sex-specific philopatry), agricultural depredation, feeding ecology, physiology of migration and sensitivity to environmental change.

## Introduction

Conservation and management of migratory wildlife is dependent upon our understanding of species-specific spatial ecology, reproductive biology and sensitivity to environmental change. Although generally abundant, some subpopulations of the Red-winged Blackbird (*Agelaius phoeniceus*) have experienced declines since 1965 [1]. Although the Red-winged Blackbird (hereafter blackbird) is one of the most studied wild birds in North America, current research priorities for this species include sex-specific philopatry, net impacts to agricultural crop production, feeding ecology during the nonbreeding season and aspects of physiology related to migration [2].

Producers of U.S. rice, corn and sunflower commodities have experienced agricultural depredation caused primarily by blackbird damage to their newly-planted and ripening crops [3, 4, 5]. Managing bird damage to agricultural crops by blackbirds within the U.S. currently involves various lethal and non-lethal approaches as part of an integrated pest management strategy [4]. Non-lethal management strategies include propane cannons, chemical repellents, decoy crops and modified agricultural practices. Such practices include changing to crops not affected by birds, synchronized planting or planting larger fields, delaying the plowing of harvested grains to provide alternative food, and herbicide and insecticide applications [4]. Lethal management strategies often include the use of avicides and trapping at sites associated with depredation. Clearly, the efficacy of each of these management strategies would be enhanced if the subpopulations, movements and food habits of blackbirds associated with agro-ecosystems could be identified and monitored.

Previous attempts at tracing movements of blackbirds associated with damage to agricultural crops involved use of fluorescent markers [6]. However, this approach and other conventional techniques such as banding are extremely limited as means of evaluating structure and movement of small passerines at continental scales [7]. Reliable applications of cost-effective methods are needed for blackbird control as are accurate evaluations of control efforts within the context of the overall conservation and management of blackbirds. The use of intrinsic markers such as naturally occurring stable isotopes of the light elements (carbon, nitrogen, hydrogen, sulfur) in animal tissues provides an additional or complimentary means of tracking migratory movements of birds and other animals that can be useful in species conservation and management [8].

Stable-carbon (*δ*^13^C) and hydrogen (*δ*^2^H) isotope ratios in feathers were previously used to delineate geographic origins among 64 blackbirds collected along a latitudinal gradient from Louisiana, USA, to Saskatchewan, Canada [9]. Stable-hydrogen isotope values from primary feathers decreased with latitude and correlated well with amount-weighted, mean growing-season *δ*^2^H for precipitation (*δ*^2^H_p_) at collection sites (*r*^2^ = 0.83). Stable-carbon isotope values further segregated those individuals feeding on C3- versus C4-based diets. That study suggested that the isotope approach could be used to investigate origins of blackbirds potentially associated with agricultural crop depredation throughout the continental Midwest. Indeed, an optimal approach to investigating origins of migratory wildlife using stable isotopes would include ground-truthed isotopic basemaps or isoscapes based upon same-year measurements of breeding and wintering subpopulations [9]. Since that early study, use of stable isotope methods to delineate origins of migratory birds in North America and elsewhere has increased tremendously [8, 10], but most studies have relied almost exclusively on feather *δ*^2^H measurements. The dietary breadth of blackbirds that includes both C3 and C4 diets, together with an understanding of the distribution and production of agricultural crops throughout the species’ breeding range, presents an opportunity for a more refined, multi-isotope approach for assignment to molt origin [11, 12, 13].

We hypothesized that feather isotopes could be used to predict the molt origin of Red-winged Blackbirds collected in spring and winter near U.S. sunflower and rice production. The interstate movements of migratory birds are fundamental to planning national and international conservation and management efforts. With regard to overabundant species and the management of agricultural depredation caused by some wild birds, an understanding of the molt origin, immigration and emigration of red-winged blackbirds collected near sunflower and rice production would enable interstate coordination of ongoing and planned damage management (e.g. application of non-lethal chemical repellents throughout the rice-growing region of the Mid-south) [14]. The objectives of this study were to use *δ*^2^H, *δ*^13^C and *δ* ^15^N measurements to better understand the molt origins, and the C3 and C4 food habits of blackbirds near sunflower production in the Upper Midwest and rice production in the Mid-South of the United States. We expected *δ*^2^H and *δ*^13^C measurements to provide the greatest resolution for spatial assignments but included *δ*^15^N analyses because this isotope is also sensitive to land-use practices and other anthropogenic factors that might vary in intensity over our study area [15, 16].

We collected adult birds during spring and winter to investigate how well these three isotopes could discriminate among molt origins and we established feather isoscapes based upon *δ*^2^H and *δ*^13^C values. We then modeled feather isoscapes using either geostatistical approaches or known crop distributions and validated these models by assigning a hold-out sample of known-origin individuals to their most probable molt origins. We further derived expected proportions of C3 and C4 diets based upon feather *δ*^13^C values alone. These fundamental, yet previously-unknown spatial relationships can be used to prescribe and implement conservation and management efforts for wild birds, including Red-winged Blackbirds associated with agro-ecosystems.

## Methods

### Feather Sampling

The breeding distribution of Red-winged Blackbirds includes marsh and upland habitats from southern Alaska and central Canada to Costa Rica, and from California to the Atlantic Coast and West Indies [2]. Blackbirds winter in southeastern Alaska, along the U.S.–Canadian border, throughout their breeding range to south Baja, California, the north Pacific slope of Mexico, the Gulf Coast and Florida. The spatial extent of blackbird research and management regarding agricultural depredation, however, is usually defined by state or provincial boundaries.

We collected 247 after-second-year (ASY), territorial male blackbirds from 5–29 June 2012 adjacent to sunflower or corn fields in Minnesota, Montana, North Dakota and South Dakota. Forty-two additional, ASY (male) blackbirds were collected from 3–7 June 2013 to supplement the Minnesota collection. We collected 372 ASY, male blackbirds from 9–19 January 2013 adjacent to rice fields in Arkansas, Louisiana, Mississippi, Missouri and Texas. With few exceptions (e.g. Texas), we planned collections for <20 birds per site and 5–12 sites per state, and sites were selected ≥5 km from nearest sites to maximize the geographic scope of our study. We used a handheld GPS unit to record the latitude and longitude of each collection site for subsequent spatial analyses. The collection and use of blackbirds for this study were approved by the USDA, National Wildlife Research Center’s Institutional Animal Care and Use Committee (Fort Collins, CO; QA-1987, S.J. Werner- Study Director).

With regard to the ethical justification for our sampling method, our lethal blackbird collections were necessary to capture the spatial extent and the requisite spatial independence among sample collections associated with the objectives of this national study. Our bird collections were authorized by Scientific Collection Licenses in each of nine states and a Federal Scientific Collecting Permit issued by the U.S. Fish and Wildlife Service. We collected <20 birds per site and sites were separated by ≥5 km. Because the Red-winged Blackbird is perhaps the most abundant wild bird in North America [2, 17], our collection of 661 blackbirds likely had no effect on the spring, winter and sometimes overabundant populations of blackbirds associated with sunflower and rice production in the Upper Midwest and the Mid-South of the United States. For relative context, the population of Red-winged Blackbirds in the U.S. was estimated to be 120 million in 2013 [18].

We removed one wing from each collected bird [9]. Wing samples were stored in labeled paper bags and frozen until shipped to the Stable Isotope Laboratory of Environment Canada in Saskatoon. We removed a single outer primary (P8 or P9) from each wing for analysis. The second and subsequent prebasic, or post-nuptial molt occur approximately one year after the first prebasic molt (i.e. 45–60 days after fledging, thereafter annually) and the average date for the full development of new primary feathers is October 1 (P7–P9) in this species [19]. Thus, our stable isotope data for each site are based upon single *δ*^2^H, *δ*^13^C and *δ*^15^N values for each individual bird collected at that site [9].

### Stable Isotope Analysis

All feathers were cleaned of surface oils in 2:1 chloroform:methanol solvent rinse and prepared for *δ*^2^H, *δ*^13^C and *δ*^15^N analyses at the Stable Isotope Laboratory (Environment Canada, Saskatoon, Canada). The *δ*^2^H value of the non-exchangeable hydrogen in feathers was determined using previously-described methods [20] and two calibrated keratin hydrogen-isotope reference materials (CBS: -197.‰, KHS: -54.1 ‰). Hydrogen isotopic measurements were performed on H_2_ gas derived from high-temperature (1350°C) flash pyrolysis of 350 ± 10 μg feather subsamples and keratin standards using continuous-flow isotope-ratio mass spectrometry. Measurement of the two keratin laboratory reference materials, corrected for linear instrumental drift, were both accurate and precise with typical within-run (*n* = 5) SD values of <2 ‰. All results are reported for non-exchangeable H expressed in typical delta (*δ*) notation, in units per mil (‰), and normalized on the Vienna Standard Mean Ocean Water–Standard Light Antarctic Precipitation (VSMOW-SLAP) standard scale.

For *δ*^13^C and *δ*^15^N analyses, 0.5–1.0 mg of feather material was combusted online using a Eurovector 3000 (Milan, Italy– www.eurovector.it) elemental analyzer. The resulting CO_2_ was separated by gas chromatography (GC) and introduced into a Nu Horizon (Nu Instruments, Wrexham, UK– www.nu-ins.com) triple-collector isotope-ratio mass-spectrometer via an open split and compared to a pure CO_2_ or N_2_ reference gas. Stable nitrogen (^15^N/^14^N) and carbon (^13^C/^12^C) isotope ratios were expressed in *δ* notation, as parts per thousand (‰) deviation from the primary standards, atmospheric AIR and Vienna Pee Dee Belemnite (VPDB). Using previously calibrated internal laboratory standards (powdered keratin [BWB II: *δ* ^13^C = -20.0*‰*, *δ*^15^N = -14.1‰ and gelatin: *δ* ^13^C = -13.6*‰*, *δ*^15^N = -4.7‰]) within run (*n* = 5), precision for *δ*^15^N and *δ*^13^C measurements was ~ ± 0.15‰. Our master dataset (feather isotopes for each of 661 ASY blackbirds with collection-site location data) has been made publically available for subsequent investigations [S1 Table].

### Statistical Analysis

We used discriminant function analysis to predict collection states from feather *δ*^2^H, *δ*^13^C and *δ*^15^N values (SAS 9.2). The STEPDISC procedure of SAS was used to identify the best-fit model among all possible combinations of *δ*^2^H, *δ*^13^C and *δ*^15^N data. We used 95% confidence intervals and linear regression, and descriptive statistics (mean, SE, range) to analyze and summarize stable isotopes among states, respectively.

### Molt Origins of Red-winged Blackbirds Collected in Spring

Prior to feather isoscape creation, we randomly split data from 40 known-origin spring collection sites into two subsets. One subset was used for model creation (*n* = 30 sites) and the remaining ten sites were used for independent validation of our isoscapes. We then created isoscapes from the model creation set using either ordinary kriging or regression combined with spatial interpolation of model residuals as detailed below.

#### δ^2^H Isoscape

We used ordinary point kriging to spatially interpolate *δ*^*2*^H values of feathers from 208 individual birds collected at 30 independent sites that were randomly selected for model development. We examined alternative anisotropic and isotropic variogram models and selected amongst alternative models by attempting to minimize root-mean-squared errors while not over-fitting the data. Model parameters were optimized using iterative cross-validation. We then used 81 birds collected at the ten independent sites (see above) for model validation. We derived two competing isoscapes and used regression analysis to compare observed *δ*^*2*^H values of feathers from 81 known-origin birds (i.e. validation samples) against predicted *δ*^*2*^H values from each of the competing isoscapes. Finally, we selected among the two competing isoscapes using a combination of Akaike’s Information Criterion with sample size adjustment (AIC_c_) [21], and residual diagnostics such as quantile-quantile plots and residual versus fitted plots. All kriging and spatial interpolation (below) was conducted in ArcGIS v 10.1 (ESRI, Redlands, CA) using the Geostatistical Analyst™ extension.

#### δ^13^C Isoscape

We used a combination of linear modeling and spatial interpolation of model residuals to create spatially-explicit *δ*^13^C isoscapes. Blackbirds have varying access and reliance upon agricultural crops for a portion of their diet, which can then be reflected in the isotopic composition of their feathers. In addition, crops planted in the U.S. Midwest include species using both C3 and C4 photosynthetic pathways, which are known to differ in the degree to which they incorporate ^13^C [22, 23] and therefore could be expected to alter the relative isotopic composition of feathers. We therefore compared *δ*^13^C values of our feather samples at a given collection site to county-level data on acreage of C3 and C4 agricultural crops derived from the United States Department of Agriculture’s Agriculture Statistics Service (http://quickstats.nass.usda.gov/). Acreage estimates of planted and harvested corn, millet, rice, sorghum and sunflower (oil, confection) were obtained for the 2012 and/or 2013 growing seasons. We then summed the area of all planted acres of C3 and C4 crops in each of 2012 and 2013, and divided this value by the total area of the given county. We then averaged this value between 2012 and 2013, thus providing the average proportion of the county that was planted with C3 and C4 crops. That proportion was used as a linear covariate in subsequent regression models. We performed a spatial join in ArcGIS (version 10.0; ESRI, Redlands, California) to compare national and county-based crop estimates for each sampled county to unsampled counties. In addition, *δ*^13^C values have previously been shown to be correlated with *δ*^2^H values in feathers in some ecosystems [12, 24]. Thus, we also considered *δ*^2^H as a potential explanatory variable for the *δ*^13^C values of blackbird feathers.

We created four competing regression models including main effects and interactions for the average proportion of C3 and C4 crops in the sampled county and *δ*^2^H values in feathers. We used AIC_c_ based model selection as a first step toward choosing a parsimonious model [21]. Where model uncertainty occurred, we based inference on the least parameterized model within the 'confidence set' that included variables with 85% confidence intervals that did not overlap zero to be consistent with AIC_c_ based model selection [25]. We did not use model averaging of parameter estimates as this can result in bias in the presence of relatively minor correlation [26, 27]. Since we were interested in spatially-explicit predictions, we saved the residuals of this model and spatially interpolated those values which were later added to predictions from our top models to improve estimates and reduce violations of the linear model assumptions of independence.

### Molt Origins of Red-winged Blackbirds Collected in Winter

In order to assess the origins of blackbirds collected during winter, we used a previously described spatially-explicit approach to assign birds to their putative molt origin using a Bayesian framework to compare observed *δ*^*2*^H values in feathers against prediction from calibrated feather isoscapes [28, 29, 30]. We combined data from a previous study using 64 blackbirds collected at 11 sites on a north-south transect through a large portion of the breeding range [9], with data from an additional 288 birds collected at 39 sites sampled in this study. For each collection site, we calculated sample size and mean (±SD) of feather *δ*^*2*^H values. In addition, we obtained the predicted amount-weighted mean *δ*^*2*^H value of growing-season precipitation (*δ*^*2*^H_p_) from a previous isoscape [31] for each site where known-source blackbirds were collected. We then derived a rescaling function to calibrate a feather *δ*^*2*^H isoscape (*δ*^*2*^H_f_) by bootstrap regression of 1000 randomly generated data sets in which we propagated errors associated with between-individual variance and spatially-explicit errors in the precipitation isoscape by simulating data to match site-level means and standard deviations for both feather and *δ*^*2*^H_p_ values (Table 1) [32]. This resulted in a mean rescaling function of *δ*^*2*^H_f_ = -37.45 (SD = 2.94) + 0.86 (SD = 0.04)* *δ*^*2*^H_p_. We used this equation to derive a feather isoscape.

**Table 1:** Sample size, mean and standard deviation of *δ*^*2*^H in feathers (‰) sampled along a gradient of predicted *δ*^*2*^H in precipitation (‰). *δ*^*2*^H in precipitation (and isoscape prediction errors [SD]) was derived from previous, spatially-explicit predictions [31].

Collection			*δ*^*2*^H in feathers			*δ*^*2*^H in precipitation	
Site	Latitude	Longitude	*n*	Mean	SD	Mean	SD
1^*^	30.20	-91.70	8	-53	6.9	-24	11.3
2^*^	30.40	-91.00	6	-44	12.1	-23	11.3
3^*^	32.88	-88.43	4	-62	11.3	-31	11.2
4^*^	36.78	-90.03	5	-69	9.6	-43	11.3
5^*^	38.93	-90.58	5	-61	8.4	-45	10.9
6^*^	39.02	-96.82	4	-63	13.1	-48	10.8
7^*^	40.78	-91.38	4	-54	7.4	-48	10.6
8^*^	43.28	-91.98	8	-77	5.0	-60	10.5
9^*^	43.43	-93.22	4	-71	10.2	-59	10.2
10^*^	46.75	-97.50	10	-100	11.4	-72	9.9
11^*^	51.70	-106.50	6	-135	13.6	-101	8.9
12	47.70	-96.19	11	-101	13.3	-73	9.7
13	47.56	-96.22	4	-99	6.6	-72	9.7
14	47.61	-96.19	3	-102	9.6	-72	9.7
15	47.68	-96.20	18	-96	8.6	-73	9.7
16	48.14	-96.14	10	-79	7.1	-75	9.6
17	48.18	-96.72	5	-90	4.9	-74	9.6
18	47.26	-96.36	10	-75	6.7	-69	9.8
19	45.70	-94.32	8	-73	8.3	-64	9.9
20	44.99	-95.51	9	-61	5.9	-60	10.1
21	48.32	-104.49	4	-110	8.8	-85	9.7
22	48.58	-109.12	8	-124	7.6	-90	9.8
23	48.36	-107.99	9	-122	6.8	-88	9.8
24	45.93	-108.20	7	-118	9.2	-84	10.5
25	46.29	-107.24	7	-120	7.9	-84	10.4
26	46.27	-106.83	10	-122	7.9	-83	10.4
27	46.28	-104.41	8	-122	7.5	-83	10.0
28	46.42	-104.63	3	-123	1.1	-83	10.0
29	45.70	-104.33	3	-114	5.7	-82	10.0
30	46.79	-104.14	4	-124	7.9	-84	10.0
31	46.04	-104.37	6	-115	8.5	-83	10.0
32	46.50	-100.08	5	-93	2.8	-78	10.0
33	47.25	-100.58	3	-103	2.9	-81	10.0
34	47.32	-100.54	10	-102	3.5	-81	10.0
35	47.50	-100.49	10	-104	5.0	-82	9.9
36	48.01	-101.07	1	-122		-23	
37	47.58	-101.05	3	-126	18.7	-19	5.2
38	47.59	-101.03	7	-120	7.0	-22	2.9
39	46.22	-100.04	6	-109	6.9	-20	3.6
40	46.27	-99.51	10	-115	8.0	-23	0.9
41	46.38	-99.44	7	-112	5.7	-23	3.2
42	46.50	-99.51	10	-111	7.8	-23	0.5
43	47.28	-99.51	5	-117	7.1	-25	1.4
44	44.78	-99.82	12	-105	10.2	-21	3.2
45	44.35	-100.01	3	-117	10.5	-26	6.5
46	44.80	-99.95	10	-99	9.2	-22	1.8
47	44.95	-99.92	7	-97	8.7	-22	2.1
48	44.69	-100.13	14	-90	11.2	-21	2.2
49	45.15	-100.00	4	-99	18.6	-22	2.0
50	45.53	-100.30	11	-107	11.5	-21	2.7
51	45.41	-99.94	4	-112	7.7	-22	2.8

^*^ Data from [9].

We conducted assignments to origin by applying normal probability density functions to provide a spatially-explicit assessment of the likelihood that a given feather was grown at a given location within the isoscape. This was accomplished by comparing the observed *δ*^2^H_f_ value against the isoscape predicted (as derived above) *δ*^2^H value for a given pixel within the isoscape. We parameterized the normal probability density function by treating isoscape predicted *δ*^2^H_f_ values (within every pixel) as the means against which *δ*^2^H_f_ was compared [29, 30]. In addition, we derived spatially-explicit variance estimates to include in the likelihood assessment [32]. These variance estimates were derived by pooling variance from between-individual variance (mean of SDs reported in Table 1: 8.4 *‰*), spatially-explicit prediction errors of a previous isoscape [31] and uncertainty in the recalibration equation from the 1000 bootstrap estimates (above). Combined, these pooled estimates resulted in spatially-explicit errors, ranging from 12.0–15.6 *‰*. Applying the normal probability density function resulted in one map of posterior probabilities for each bird in the sample.

In order to assign each bird to likely origins, we used a 2:1 odds ratio of being “correct” versus “incorrect” by selecting the upper 67% of the probability densities and recoded these as “likely” origins [28, 33]. If a collection site for an individual fell within the region defining those coded as “likely”, the individual was classified as a non-migrant and was removed from further analyses. The remaining birds in the analysis were categorized as migrants, and were depicted separately as birds that originated from north versus south of the sampling locale depending on whether the observed *δ*^2^H_f_ was less than (north) or greater (south) than the value predicted from the calibrated isoscape at the collection site. Finally, we applied our algorithm to assign the 81 birds sampled at ten independent sites to validate our assignment accuracy.

We used a permutation-based multivariate analysis of variance (perMANOVA) [34] to compare the stable isotope composition (*δ*^13^C, *δ*^15^N) of feathers between birds classified as resident, migrants from north of the collection sites, and migrants from south of the collection sites based on the designations derived above. We did not include *δ*^2^H values in this multivariate comparison, because our geospatial assignment approach would result in *defacto* differences. We conducted perMANOVA based on 999 permutations of Euclidean distances using the vegan R package [35]. We used permutation-based methods because a preliminary inspection of our data suggested that they did not meet the required multivariate normality (Shapiro-Wilk test W = 0.94, p < 0.001) and homogeneity of variances assumptions (Fligner-Killeen tests; *δ*^13^C χ^2^ = 15.06, df = 2, p < 0.001; *δ*^15^N χ^2^ = 12.82, df = 2, p < 0.001) required for parametric MANOVA tests. Geospatial assignments to origin were conducted using customized functions for the ‘raster’ package [36]. All analyses were conducted in the R (v. 3.2.1) statistical computing environment [37].

## Results

### Interstate Comparison of Feather Isotopes

**Fig 1:**
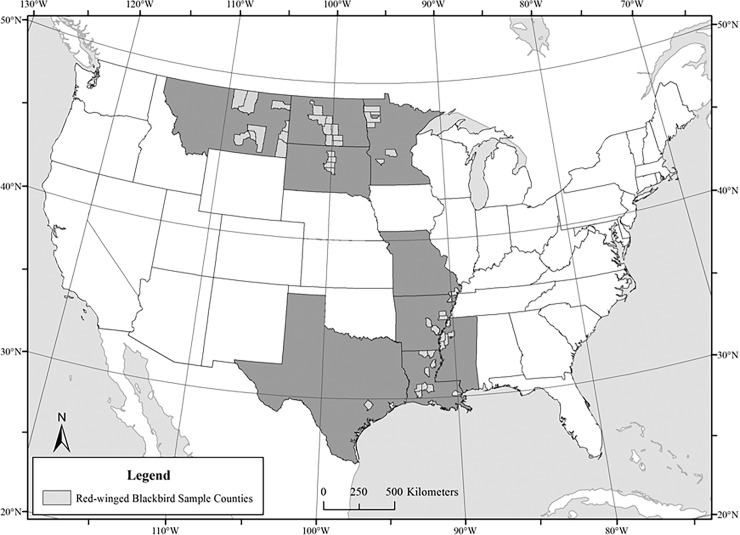
Geographic range of sample collection sites (i.e. counties) used to develop multi-isotopic depictions of origin for Red-winged Blackbirds collected in spring and winter near U.S. sunflower and rice production. Source data for map features were from ESRI Data & Maps for ArcGIS (http://www.esri.com/data/data-maps; accessed 23 July 2016).

We observed a considerable range in *δ*^2^H (˗153.0 to ˗20.3 ‰), *δ*^13^C (˗32.2 to ˗10.1 ‰) and *δ*^15^N (4.7 to 16.7 ‰) values in feathers of blackbirds (Table 2) [S1 Table]. The range of latitude and longitude among collection sites was 29.482–48.575° N and 89.572–109.124° W (Fig 1). Feather *δ*^2^H values were correlated with the latitude (*r* = ˗0.83; adjusted *r*^*2*^ = 0.67, *P* < 0.0001) and longitude (*r* = 0.73; adjusted *r*^*2*^ = 0.52, *P* < 0.0001) of collection sites. Feather *δ*^13^C values were also inversely related to latitude (*r* = ˗0.18; adjusted *r*^*2*^ = 0.03, *P* < 0.0001) and positively related to longitude (*r* = 0.17; adjusted *r*^*2*^ = 0.03, *P* < 0.0001). Values of *δ*^15^N were inversely related to both latitude (*r* = ˗0.22; adjusted *r*^*2*^ = 0.03, *P* < 0.0001) and longitude of collection sites (*r* = ˗0.10; adjusted *r*^*2*^ = 0.01, *P* = 0.0008). We observed the greatest differences between states in *δ*^2^H_f_ values. Non-overlapping, 95% confidence intervals suggested that *δ*^2^H_f_ values were different for feathers collected in Texas, Minnesota, South Dakota, North Dakota and Montana (Table 2).

**Table 2:** Interstate comparisons of stable isotopes in after-second-year Red-winged Blackbirds. For each of *δ*^2^H, *δ*^13^C and *δ*^15^N (‰), non-overlapping 95% confidence intervals are indicated by unique letters in the last column.

Isotope/state	mean	SE	*n*	Min.	Max.	Lower CI	Upper CI	95% CI
*δ*^2^H								
Texas	-40.3	1.0	85	-70.1	-20.3	-42.3	-38.2	a
Mississippi	-53.8	1.0	69	-82.8	-37.6	-55.8	-51.8	b
Louisiana	-55.5	1.4	85	-94.6	-31.0	-58.4	-52.7	b
Missouri	-56.1	2.0	58	-124.3	-36.8	-60.2	-52.0	b,c
Arkansas	-64.7	2.9	75	-153.0	-32.2	-70.4	-59.0	c
Minnesota	-85.2	1.8	78	-123.7	-50.7	-88.8	-81.6	d
South Dakota	-101.1	1.6	65	-129.5	-75.9	-104.3	-97.9	e
North Dakota	-109.8	1.2	77	-144.0	-89.8	-112.2	-107.5	f
Montana	-120.2	1.0	69	-136.4	-101.7	-122.1	-118.2	g
*δ*^13^C								
Missouri	-18.2	0.6	58	-26.6	-10.1	-19.4	-17.1	a
Texas	-19.4	0.4	85	-24.9	-13.0	-20.1	-18.7	a
Arkansas	-19.8	0.6	75	-28.9	-11.0	-21.0	-18.5	a,b
Minnesota	-21.4	0.4	78	-25.9	-12.2	-22.2	-20.6	b,c
South Dakota	-21.7	0.3	65	-32.2	-15.8	-22.4	-21.0	b,c
Louisiana	-22.1	0.6	85	-29.6	-11.1	-23.2	-21.0	b,c,d
Mississippi	-22.3	0.5	69	-27.3	-10.9	-23.4	-21.3	c,d
Montana	-22.5	0.4	69	-26.3	-12.9	-23.3	-21.7	c,d
North Dakota	-23.2	0.3	77	-26.6	-14.0	-23.8	-22.6	d
*δ*^15^N								
Texas	12.1	0.2	85	7.3	15.9	11.8	12.4	a
North Dakota	10.0	0.2	77	4.7	14.7	9.7	10.3	b
Montana	10.0	0.2	69	6.5	15.7	9.6	10.3	b
Louisiana	9.9	0.1	85	6.9	15.7	9.7	10.2	b
South Dakota	9.9	0.2	65	6.8	13.9	9.5	10.2	b
Missouri	9.7	0.2	58	7.2	16.7	9.2	10.2	b
Minnesota	9.6	0.2	78	6.7	14.0	9.3	10.0	b
Mississippi	9.6	0.1	69	7.4	13.8	9.3	9.9	b
Arkansas	9.5	0.2	75	6.7	16.1	9.2	9.8	b

When considering only birds collected in spring, 56% of birds were classified to their collection state on the basis of *δ*^2^H and *δ*^13^C alone (Table 3). Although this percentage decreased to 44% when the combination of *δ*^2^H, *δ*^13^C and *δ*^15^N was used, a stepwise discriminant analysis indicated that the inclusion of feather *δ*^2^H, *δ*^13^C and *δ*^15^N values provided the best-fit model for the classification of collection states (Wilks’ Lambda *F*_24,1885_ = 72.83, *P* < 0.0001). Thus, the latitude (adjusted *r*^*2*^ = 0.68, *P* < 0.0001) and longitude (adjusted *r*^*2*^ = 0.57, *P* < 0.0001) of collection sites were related to the combination of *δ*^2^H, *δ*^13^C and *δ*^15^N values for blackbird feathers.

**Table 3:** Discriminant function classifications among stable isotope combinations for after-second-year Red-winged Blackbirds.

	Percent of Birds Classified as Having Originated in the Same State as their Collection Site		
Stable Isotope	All Samples (*n* = 661)	Spring Samples (*n* = 289)	Winter Samples (*n* = 372)
H	37	54	35
C	17	33	27
N	22	29	37
HC	42	56	44
HN	40	54	39
CN	23	31	41
HCN	44	56	47

### Relative Use of C3- and C4-based Foodwebs

We calculated relative percent of C3 and C4 plant-based contributions to diets of blackbirds in each state using a simple two-source (C3, C4), single-isotope (*δ*^13^C) linear mixing model. However, because we did not measure *δ*^13^C values of crops directly at each collection site, and because there can be a considerable isotopic range across plants in each of these categories [22], we considered each population of interest individually and examined the distribution of feather *δ*^13^C values. In general, the feather *δ*^13^C data conformed well to expectations of a broad C3 to C4 isotopic range reflecting the isotopic difference between C3 and C4 plants, or about -27 *‰* to -12 *‰*, respectively [23], after considering an ~1‰ isotopic discrimination between diet and feather [11]. Because these simple mixing models are sensitive to choice of endpoints (occasionally providing negative estimates of contributions for some individuals with extreme values), we instead examined the 5^th^ and 95^th^ percentiles of the distributions and, if these conformed well to the expected 15 ‰ C3 to C4 range of feather *δ*^13^C values for each population (i.e. that range expected between a pure C3 and a pure C4 diet) [23], we associated these points to the 100% C3 and 100% C4 endpoints, respectively. Any individuals falling below the 5^th^ percentile or above the 95^th^ percentile were considered to have 100% C3 and C4 diets, respectively. We determined a broad range of C4 dietary inputs among individuals within populations (Table 4).

**Table 4:** Estimate of percent C4 contributions to diets of after-second-year Red-winged Blackbirds.

State	C4 (%)	SD	*n*	5^th^ Percentile (‰)	95^th^ Percentile (‰)
δ^13^C					
Arkansas	50.2	33.9	75	-27.9	-12.3
South Dakota	48.8	24.2	75	-27.1	-16.1
Missouri	47.4	32.2	60	-24.7	-11.6
Texas	45.7	31.8	85	-23.9	-13.9
Louisiana	36.9	30.7	85	-28.2	-11.8
North Dakota	27.9	25.4	82	-26	-16.1
Mississippi	27.5	28.9	69	-26.5	-12.2
Montana	27.4	26.0	76	-25.7	-14.2
Minnesota	26.2	28.3	78	-25.6	-13.8

### Molt Origins of Red-winged Blackbirds Collected in Spring

#### δ^2^H Isoscape

**Fig 2:**
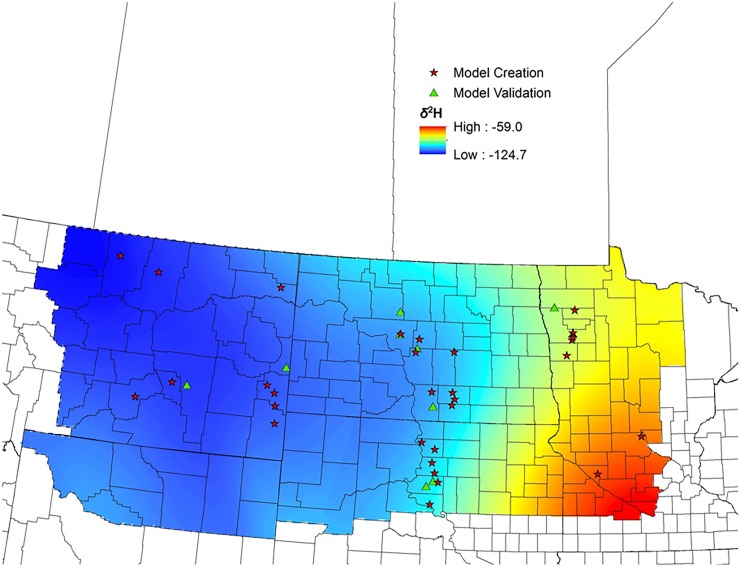
Predicted stable-hydrogen isotope ratio of Red-winged Blackbird feathers (*δ*^2^H) in the U.S. Upper Midwest derived from geostatistical analysis (see Methods) of *δ*^2^H from 208 after-second-year birds collected at 30 independent sites.

**Fig 3:**
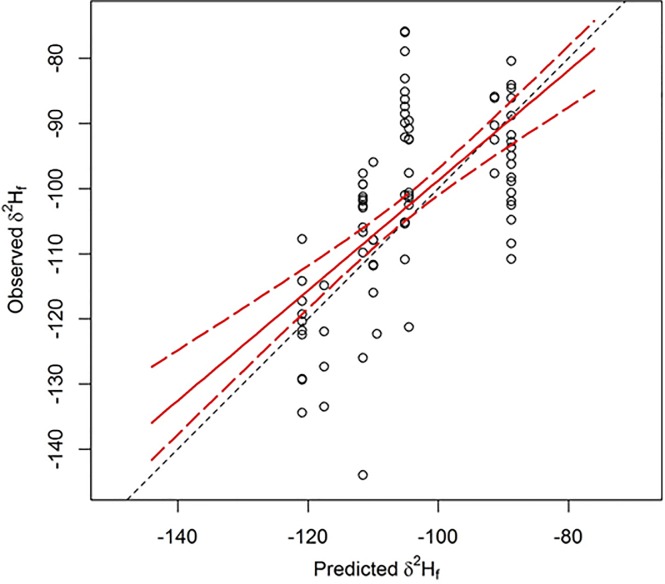
External validation of our *δ*^2^H isoscape model based on comparing observed *δ*^2^H in feathers of 81 birds from 10 independent sites against model predicted *δ*^2^H for feathers at the same 10 collection sites. Dashed black line represents 1:1 correspondence, solid red line depicts ordinary least squares fit between axes, and curved red dashed lines are 85% confidence intervals for the relationship between observed and predicted *δ*^2^H.

After exploring alternative formulations for sample semi-variograms, we arrived at two models which fit the data well and which we considered competitive models for isoscape creation. One model was an anisotropic Gaussian model, while the other was an isotropic stable variogram model. We extracted predictions from kriged surfaces created for each of these two models at locations with our validation samples. Regression of *δ*^2^H_f_ values from known-origin feather samples against the predicted values resulted in the isotropic stable variogram model being heavily favored, receiving 95% of the support based on AIC_*c*_ model weights. The selected model had a lag distance of ~80.6 km, a range of ~718 km, a nugget of 132.01, and a partial sill of 380.20. The resulting isoscape predicted an isotopic gradient that was most enriched in ^2^H (i.e. least negative values) in the southeastern portion of our study area, and most depleted in ^2^H in the northwest (i.e. most negative values; Fig 2). The top model explained ~36% of the variance in the withheld validation data, and 85% confidence intervals for the slope (β = 0.80, SE = 0.12) overlapped the 1:1 correspondence line over much of the isotopic gradient, but was biased for birds whose feathers were more negative than ~ -105*‰* (Fig 3).

#### δ^13^C Isoscape

**Fig 4:**
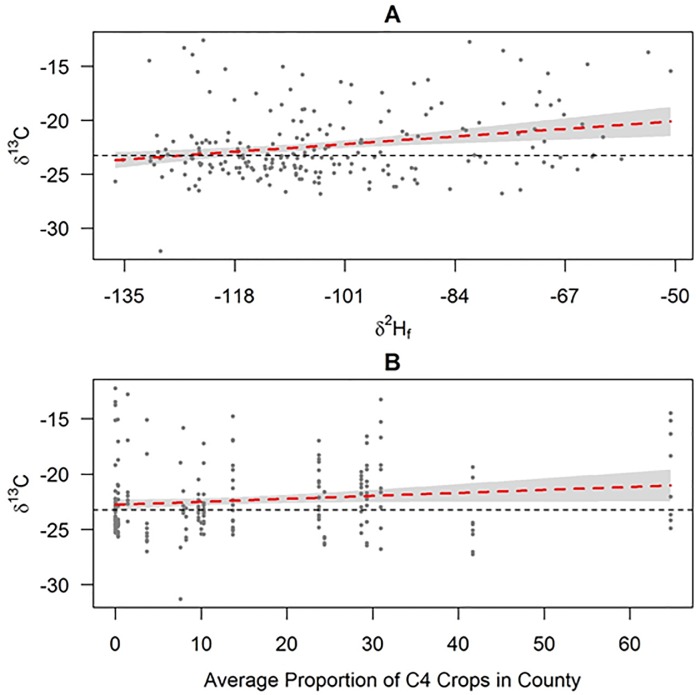
Partial regression plots for the relationship between *δ*^13^C and A) stable-hydrogen isotope ratio of feathers (*δ*^2^H) and B) the average proportion of the sampled county that was planted to C4 crops (2012 & 2013). Partial plots were created using model parameters reported above while setting other covariates constant at their median value. Heavy dashed red line represents predicted relationship (± 85% confidence intervals in gray shading). Dashed horizontal black line represents median *δ*^13^C.

Based on AIC_c_ model selection and elimination of models with “pretending parameters” [38], the top model explaining variation in *δ*^13^C of blackbirds collected in spring included an intercept (β = -18.28, SE = 1.88), a slope for *δ*^2^H (β = 0.04, SE = 0.02) and the average proportion of C4 crops in the county in which the bird was collected (β = 0.03, SE = 0.02). This model explained ~11% of the variation in *δ*^13^C, and suggests that birds collected in regions of the study area that were most depleted in ^2^H (most negative *δ*^2^H_f_ value) were also the most depleted in ^13^C (Fig 4A), and that feather *δ*^13^C was also positively related to the average proportion of C4 crops grown in the sampled county (Fig 4B). While there was a statistical relationship between feather *δ*^13^C values and our selected parameters, the resulting isoscape failed to adequately predict feather *δ*^13^C values for our validation samples, with an intercept that differed substantially from zero (β = -18.38, SE = 4.40), and a slope that departed substantially from 1 (β = 0.17, SE = 0.20).

### Molt Origins of Red-winged Blackbirds Collected in Winter

**Fig 5:**
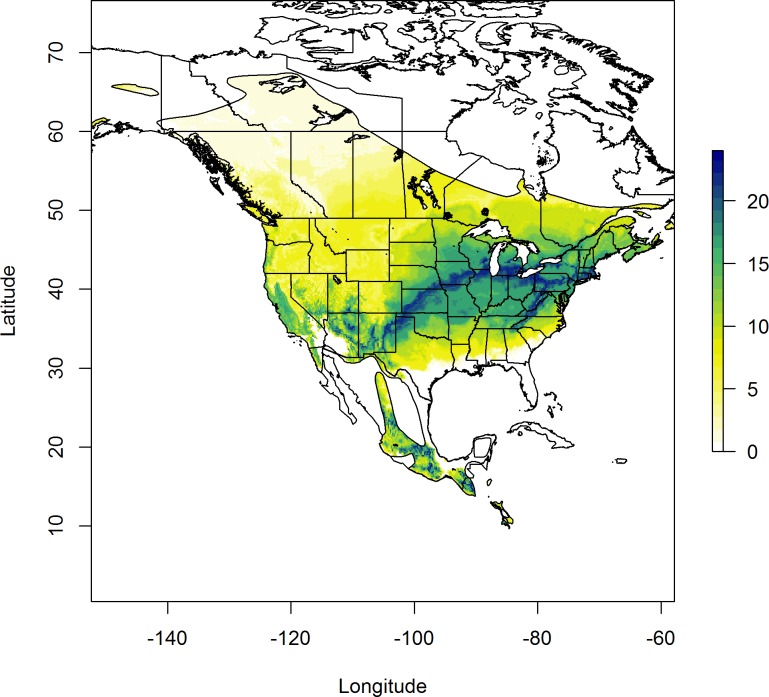
Geographic distribution of assigned origins of 32 Red-winged Blackbirds collected during the winter of 2013 that were assigned to origins north of their collection sites in Arkansas, Louisiana, Mississippi, Missouri and Texas (see Methods). Legend colors represent the number of birds within the sample that were isotopically consistent with pixels of the same color on the map representing a plausible origin at 2:1 odds. Birds were assigned to their putative origins using a likelihood based assignment algorithm to compare observed *δ*^2^H in feathers against predicted values from rescaling of a previous precipitation *δ*^2^H isoscape [31].

**Fig 6:**
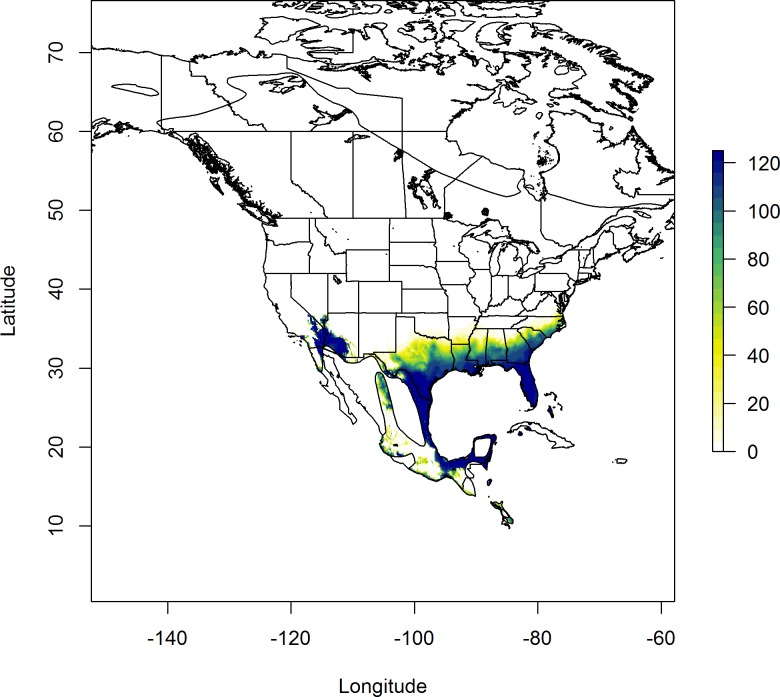
Geographic distribution of assigned origins of 125 Red-winged Blackbirds that were assigned to origins south of their collection sites in Arkansas, Louisiana, Mississippi, Missouri and Texas (see Methods). Legend colors represent the number of birds within the sample that were isotopically consistent with pixels of the same color on the map representing a plausible origin at 2:1 odds. Birds were assigned to their putative origins using a likelihood based assignment algorithm to compare observed *δ*^2^H in feathers against predicted values from rescaling of a previous precipitation *δ*^2^H isoscape [31].

The validation of our spatially-explicit assignment to the *δ*^2^H_f_ isoscape resulted in 62 individuals from our validation sample of 81 birds (~77%) being accurately assigned to the site at which they were collected in spring. We therefore applied this model to assign birds collected in winter to their putative molt origins. We collected 372 blackbirds on the wintering grounds, 215 of which were assigned to likely origins that overlapped their sampling locale and were thus considered residents. The remaining 157 wintering samples were considered migrants, 32 of which had putative origins north of their collection sites (Fig 5). The likely origins of these migrants were centered on a band from northeastern New Mexico, through the Texas panhandle into Kansas through to Iowa, and isotopically similar areas of Michigan and the Appalachians (Fig 5). While the majority of the northern migrants were consistent with low latitudes in the U.S., a few individuals were consistent with having originated as far as the Northwest Territories (*n* = 1) and Alaska (*n* = 3; Fig 5). Birds classified as having originated from areas south of their collection site were all assigned high likelihoods of originating along areas adjacent to the Gulf of Mexico (Fig 6). Southern origin birds were also consistent with isotopically similar areas of south eastern California and Arizona (Fig 6).

**Fig 7:**
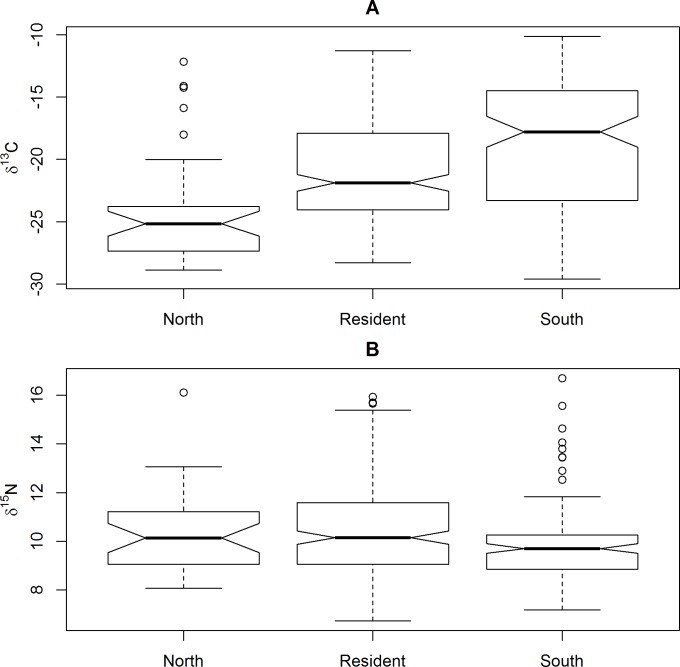
Boxplots displaying variation in A) *δ*^13^C and B) *δ*^15^N between birds classified as local residents versus migrants from north or south of their collection site on the basis of geospatial assignments to origin from analysis of *δ*^2^H_f_ (see Methods and Results). Dark solid line represents the median, box indicates the inter-quartile range, whiskers are 1.5 time the inter-quartile range, and dots indicate extreme values. Lack of overlap in notches suggests a statistical difference in the means.

Migration status (north, resident or south) explained approximately 10.1% of the variation in the isotopic composition (*δ*^13^C, *δ*^15^N) of blackbirds feathers (F_2,369_ = 20.69, *P* < 0.001). Graphical examination of the data suggests that the multivariate difference was primarily due to variation in feather *δ*^13^C values, with migrants from the north tending to be substantially more depleted in ^13^C than either residents or migrants from south of their collection sites (Fig 7A). In contrast, there was little evidence for any biologically meaningful differences in feather *δ* ^15^N values between putative migrants (south or north) and residents (Fig 7B).

## Discussion

It was previously suggested that the feather isotope approach could be used to investigate the origins of Red-winged Blackbirds potentially associated with agricultural crop depredation throughout the continental Midwest [9]. When considering only birds collected in June, 56% of blackbirds in this study were classified to their collection state on the basis of *δ*^2^H and *δ*^13^C alone. The spatially-explicit assignment of the 81 birds used to validate our *δ*^2^H isoscape resulted in relatively high rates (~77%) of accurate assignment to collection states. Eighty percent of their 64 samples was classified to their mid-May–mid-July collection sites using *δ*^2^H and *δ*^13^C and values [9]. This percentage decreased to 64% when only *δ*^2^H values were used, indicating that *δ*^13^C provided a useful additional means of delineating origins of blackbirds [9].

Our extensive analyses of primary feathers from Red-winged Blackbirds collected in spring and winter indicated a broad isotopic range generally consistent with latitudinal variation in origins and use of diets based on carbon spanning a range of C3 to C4 photosynthetic pathways. These attributes predisposed this species as a good candidate for the forensic evaluation of both geographical origin and use of agricultural crops during the post-breeding molt. Our results confirm the utility of the isotope approach to investigating movements and ecology of blackbirds and other species associated with agriculture in North America and elsewhere. However, the complex nature of agricultural landscapes, especially those involving the juxtaposition of C3 and C4 crops combined with the possibility that blackbirds may disperse from the breeding locations to other areas in order to molt, makes the precise modeling of blackbird origins extremely challenging. Nonetheless, we have demonstrated that the combination of stable isotope measurements of feathers from blackbirds collected on their wintering grounds can provide important information on the structure of wintering populations, migratory connectivity and ultimate indices of crop and other dietary sources.

Although we observed a considerable range in *δ*^2^H, *δ*^13^C and *δ*^15^N values in feathers of blackbirds, our stepwise discriminant analysis indicated that the inclusion of each isotope value provided the best-fit model for the classification of collection states. Thus, combined analyses of *δ*^2^H, *δ*^13^C and *δ*^15^N values can be used for subsequent investigations of interstate, national and international movements, migration, and spring and winter habits of migratory birds and other wildlife. Moreover, conservation and management efforts can now be planned and implemented with an understanding of the subpopulations, movements and diets of wildlife at appropriate spatial scales.

The large isotopic difference between C3 (e.g. rice, sunflower) and C4 (corn, millet, sorghum) crops provided a convenient *δ*^13^C marker of blackbird use of the C4 crops. However, non-agricultural (i.e. natural) diets would presumably be mostly C3 and so the relative amount of C3 crops to diets is less readily inferred from isotopic measurements alone. Our estimate of the maximum use of C4 crops during molt was 50% for wintering blackbirds collected in Arkansas. This was followed closely by birds from South Dakota, Missouri and Texas. Relatively low (~27%) C4 inputs were estimated for diets of blackbirds collected in Missouri, Minnesota and North Dakota. Additional analyses are planned for parsing the post-molt dispersal of blackbirds collected in spring and winter relative to C3- and C4-based foodwebs.

Stable-nitrogen isotope measurements provided the least information in our samples with most populations exhibiting mean values in the range of 10‰. The exception was Texas at 12‰ and this may reflect the more arid conditions in that region in 2013. Plant δ^15^N values can be modeled based on climate variables such as rainfall amounts that are known to influence plant metabolism [39] but this isotope is inherently more difficult to model as an isoscape due to numerous other factors such as agricultural nutrient inputs, anthropogenic landscape changes and soil modifications [15, 16]. Generally speaking, we can associate high δ^15^N values in aquatic organisms with human-dominated landscapes under heavy nitrogen loading [40] and high δ^15^N values in terrestrial organisms with arid regions [41]. However, the nutrient source of N in water and microbial processes such as denitrification can complicate characterization of the N isotopic baseline [42]. Additionally, because trophic position strongly influences animal tissue δ^15^N values, tracing organisms to δ^15^N isoscapes requires a good understanding of isotopic discrimination. The relative constant feather δ^15^N values we found suggest that the agricultural landuse across regions approached maximum N fertilizer inputs and levels of intensity. This, in turn, resulted in low discrimination power for this isotope with the exception of Texas.

Stable-hydrogen isotopes from known-origin feather samples were strongly associated with predicted *δ*^2^H_f_ values. As expected, the selected model predicted greatest δ^2^H_f_ values in the southeast and least δ^2^H_f_ in the northwest portion of our study area. Thus, as has been demonstrated in numerous other studies [43, 44], δ^2^H_f_ analyses can provide reliable inferences regarding the general latitudinal origin of feathers sampled from blackbirds and this can be further refined to state delineations in some cases. The most likely complicating factor with this isotope is the potential use of groundwater for irrigation. That practice can result in overall enrichment in ^2^H compared to continental models of growing-season average precipitation and may account for the enriched feather isotope values seen in the Texas sample [45]. Our observation that 125 blackbirds collected on their winter sites associated with rice agriculture were assigned to more southern locations was unexpected. However, this could represent actual movements of birds from brackish marshes where both ^2^H and ^13^C may be relatively enriched in the foodweb. Birds that molted along the coast may have moved inland to take advantage of rice fields, consistent with previously described daily movements of ~ 75–85 km from coastal roosts to inland rice fields [46]. Alternatively, rice agriculture may involve regions of higher than expected feather *δ*^2^H and *δ*^13^C due to agricultural practices alone (especially evapotranspiration of standing surface water). Evaluation of these alternative hypotheses will require further collection of calibration samples during the breeding/molting period along the southern range edge, and/or validation using telemetry based methods.

### Future Research

Our study provides a foundation for future research into using stable isotope tracers to investigate the ecology of birds associated with agro-ecosystems. In our case, more research into the appropriate scales of model development using state crop statistics is warranted. Currently, it is not clear if isoscape modeling can be refined to the scale needed to improve assignment at the state level. Blackbirds from single sites showed a considerable range in stable isotope values. Thus, individuals may move considerable distance from their breeding sites to molt (involving different local isoscapes) and this can complicate ground-truthing or calibration studies. Currently, the most parsimonious use of the isotope data is to assign molting latitude (using *δ*^2^H measurements) and the relative use of C4 crops (using *δ*^13^C measurements) during molt. On wintering grounds, examining the proportion of local versus immigrant birds in wintering flocks has considerable potential for devising more refined strategies for managing blackbirds in an agricultural environment. In addition, there is potential to examine how flock composition may change through time and in response to management prescriptions on the breeding and wintering grounds.

We recommend multi-isotopic approaches to ecological investigations, including wildlife response to management actions and environmental change, vertebrate pest-agronomic associations, post-molt dispersal, biology of hatching-year and after-hatching-year blackbirds and the apparent paucity of natal philopatry in female Red-winged Blackbirds [47, 48]. For example, feather δ^2^H and *δ*^13^C isoscapes can support field investigations regarding the conservation and management of blackbirds. Field efficacy trials of non-lethal chemical repellents can be enhanced by understanding the movements of blackbirds following repellent applications for the protection of agricultural crops [14]. Similarly, investigations regarding the net impacts to agricultural crop production, feeding ecology during the nonbreeding season and aspects of physiology related to migration [2] can be enhanced by an understanding of the movements and habits of blackbirds at appropriate spatial scales.

## Supporting Information

S1 Table **Werner SJ, Hobson KA, Van Wilgenburg SL, Fischer JW.** Multi-isotopic (*δ*^2^H, *δ*^13^C, *δ*^15^N) feather isotopes for red-winged blackbirds associated with agro-ecosystems. Figshare 2016; https://dx.doi.org/10.6084/m9.figshare.3860772.
(XLS)
